# A Qualitative Exploration of the Practice Learning Experiences of Third-Year Nursing Students: Challenges and Opportunities

**DOI:** 10.1155/nrp/1712974

**Published:** 2025-11-29

**Authors:** Elizabeth Mueke Kiilu, Debbie Roberts, Kate Knight, Vicky Whaley, Lizzie Carter, Julie McCartney, Jennie Brady

**Affiliations:** ^1^Department of Public Health, University of Bradford, Bradford, UK; ^2^Department of Nursing, Edge Hill University, Ormskirk, UK; ^3^Department of Nursing, University of Chester, Chester, UK; ^4^Department of Nursing, University of Bradford, Bradford, UK; ^5^Department of Nursing, University of York, York, UK; ^6^Department of Nursing, University of Lancashire, Preston, UK

**Keywords:** nursing clinical practice learning, nursing practice-based learning, nursing student experiences, proficiency achievement, student preparedness, supervisors/practice assessors' roles

## Abstract

**Aim:**

This study investigated the practice-based learning experiences of third-year nursing students under the Nursing and Midwifery Council (NMC) Future Nurse Standards (2018). The study identified enablers and barriers to proficiency achievement and explored how these experiences inform their preparation for roles as registered nurses and future practice supervisors.

**Background:**

The study focused on nursing students who began their undergraduate programs in September 2020, the first cohort under the NMC Future Nurse Standards (2018). Conducted during the COVID-19 pandemic, the research explored clinical practice learning experiences across three higher education institutions in England, highlighting the challenges in meeting required proficiencies.

**Design:**

A qualitative approach was used, underpinned by hermeneutic phenomenology. Focus group discussions with 17 final-year nursing students were undertaken.

**Methods:**

Thematic analysis was employed to explore shared experiences of practice-based learning. Ethical approval was obtained, and data saturation ensured robust findings.

**Results:**

Five themes emerged: the physical and emotional impact of practice learning, lack of confidence in the process of assessment due to inconsisteny, being prepared for practice, the impact of the practice supervisor/assessor, and what might help. Students described the process of achieving proficiencies as exhausting and emotionally draining, with assessment inconsistencies and limited supervisor engagement contributing to stress, isolation, and reduced learning opportunities.

**Conclusion:**

While students navigated the pandemic's impact on education, practice supervisors/assessors were seen as pivotal yet overstretched or insufficiently prepared. Findings highlight the need for standardized assessments, improved supervisor training, and tailored preplacement support to enhance practice-based learning and better prepare students for registration and future supervisory responsibilities.

## 1. Introduction

This paper investigates the practice-based learning experiences of third-year nursing students who began their undergraduate programs in September 2020, the first cohort under the United Kingdom Nursing and Midwifery Council (NMC) Future Nurse Standards (2018). We explored students' experiences within the various clinical settings in the adult, child, and mental health fields. Conducted across three higher education institutions in England, the study explored enablers and barriers to meeting the NMC's required proficiencies amidst the challenges posed by the COVID-19 pandemic. These standards outline the proficiencies expected of a nurse at the point of registration and reflect the NMC's vision for the role of nurses in the twenty-first century [1]. An important aspect of the Future Nurse Standards (2018) is the expectation that nurses will teach and supervise students alongside their clinical roles. However, the current educational frameworks fall short in equipping students with the teaching and supervisory skills required, leaving them underprepared for these responsibilities [2]. Compounding this issue, practice supervisors often face high workloads and insufficient training, limiting their capacity to support students effectively. This can strain student–supervisor relationships, diminish students' sense of belonging in the clinical environment, and detract from their learning experiences [3, 4].

Nursing students commencing their studies in September 2020 were the first to follow the NMC Future Nurse Standards (2018), which emphasize core competencies across all nursing fields and care settings. The timing of this cohort's education coincided with the COVID-19 pandemic, greatly disrupting health care and education. The hybrid nature of the educational experience, distance learning followed by on-site clinical practice, was a significant factor in shaping student experiences. Student clinical supervision was conducted through both one-to-one sessions and group settings for a duration of approximately 5 months. Many students began their programs online, with limited clinical placements, only resuming practice opportunities in the second year. Consequently, students faced unique challenges in achieving the professional body's proficiencies. While the pandemic itself is not the focus of this study, it shaped the learning environment for these students.

## 2. Materials and Methods

This study explored the practice-based learning experiences of third-year nursing students under the NMC Future Nurse Standards (2018), identifying key enablers and barriers to proficiency achievement. The study answered the following research questions:

• What are the enablers and barriers to achieving the NMC Future Nurse Standards (2018)?
• What aspects of practice-based learning do students prioritize (if any)?
• How are students prepared for their roles as future practice supervisors?

By examining the above issues, this research contributes to a broader understanding of the limitations in nursing education. The findings aim to inform improvements that can better support student development, foster confidence, and ensure alignment with the competencies envisioned by the NMC Future Nurse Standards.

### 2.1. Research Design

A qualitative approach, underpinned by hermeneutic phenomenology [5], was used to explore and interpret the lived experiences of final-year nursing students. Hermeneutic phenomenology focuses on understanding how individuals make meaning of their experiences within their social and emotional contexts. This approach enabled the research team to examine not just what students encountered during practice-based learning, but how they interpreted and made sense of these experiences, particularly within the unique challenges presented by the COVID-19 pandemic. It supported a rich understanding of their emotional, social, and educational journeys, attending closely to language, emotion, and context in line with the expectations of the NMC Future Nurse Standards.

### 2.2. Sample and Recruitment

A sample of 17 students from a population of 1349 was purposefully selected to explore rich and detailed insights relevant to the study (Table 1). All participants were in their third year of study and had attended at least 1 year of clinical placement. In qualitative research, small sample sizes are appropriate as the goal is to gain in-depth understanding rather than generalize findings. This number was sufficient for qualitative analysis, as data saturation was achieved [6]. The chosen sample allowed for manageable data collection and meaningful analysis while ensuring diverse perspectives are represented within the scope of the research. A general call for expressions of interest to participate was made through the usual cohort virtual learning platform at each university. The participant information sheet was made available, and interested parties were invited to attend an online information session about the research. The same information was used at each site with students able to ask any questions related to their participation in the research.

### 2.3. Data Collection

Data were collected via online focus group discussions (FGDs) conducted between February and June 2023 across the three universities, with each university undertaking one FGD. Using Microsoft Teams, three researchers facilitated three FGDs, each involving 3–7 students. Each 60-min session was audiorecorded with participant consent, and recordings were securely stored on each university's password-protected cloud system, accessible only to the research team. Recordings will be deleted per university protocols. Each site conducted its own FGDs and initial analysis independently before combining findings to address the research questions. The topic guide for the interview questions is shown in Table 2.

### 2.4. Data Management

#### 2.4.1. Thematic Analysis Process

Each site conducted one FGD and independently analyzed the data using Braun and Clarke's six-phase process of thematic analysis [7]. The process began with manual transcription of the audio recordings by 4–6 appointed members of the research team at each site. In Phase 1, researchers familiarized themselves with the data by reading and re-reading transcripts to immerse themselves in the content and gain a deep understanding of participants' experiences. Phase 2 involved generating initial codes by systematically identifying meaningful features across the entire dataset. In Phase 3, the team began searching for themes by collating related codes into broader, interpretable categories. Phase 4 required a thorough review of themes, refining and combining them where necessary to ensure coherence and relevance to the coded data and the full dataset. In Phase 5, themes were clearly defined and named, ensuring that each theme captured the essence of the participants' narratives. Finally, in Phase 6, the team produced a report of the final coded themes, grounding them in illustrative quotes and linking them back to the research questions and objectives. This iterative approach allowed researchers to listen closely, identify emotionally significant and thematically relevant content, and transcribe key excerpts in detail. Timestamps and anonymization were applied throughout to ensure traceability and participant confidentiality.

#### 2.4.2. Generation of Themes

Each site generated initial themes independently before collaborating to refine and consolidate them. These thematic schemas were shared with two independent critical friends (experienced practitioners external to the research team) who reviewed the data for additional or cross-cutting themes across the three sites. Critical friends play an important role in validating and enhancing the thematic analysis [8]. While traditionally involved in curriculum work, their role here added a layer of independent review. Each site shared their thematic frameworks and selected transcripts with the critical friends, who identified cross-cutting patterns and offered insights that enriched interpretation and reduced potential bias. This collaborative, multilayered approach ensured that the data were analyzed rigorously and represented participants' lived experiences authentically.

#### 2.4.3. Data Saturation and Credibility

Saturation was reached after three FGDs involving 17 final-year nursing students across diverse fields and institutions. No new themes emerged after the third discussion, with key themes and subthemes consistently echoed across participants. The varied geographical and professional backgrounds of the students contributed to the richness and representativeness of the findings. The trustworthiness of the analysis was strengthened through multiple strategies: methodological triangulation across the three sites, analyst triangulation via independent coding, and regular team discussions. A transparent audit trail was maintained, and intercoder agreement was used to align theme development. Member checking was achieved by sharing emerging findings and thematic summaries with some participants to confirm accuracy and resonance with their experiences. These strategies ensured that participants' voices were accurately represented. They also helped researchers remain aware of and critically reflect on their own assumptions, positionalities, and potential influence throughout the research process. Member checking involved sharing emerging themes with participants to confirm the accuracy and resonance of interpretations. Reflexivity was maintained through regular team discussions, which promoted transparency and minimized the risk of researcher bias.

### 2.5. Ethical Considerations and Approval

Ethical approval for the study was first obtained by the lead investigator at Site 1 (Ethics Committee Approval E1021, granted 07.10.22, University of XXX). This approval was shared with the other participating universities, each of which secured its own ethical approval before data collection commenced. Verbal informed consent was audiorecorded before participation. Identifiable information was anonymized in transcriptions, and students could withdraw up to 24 h before the beginning of the interviews without consequences.

## 3. Results

The emergent themes identified by the researchers and critical friends are presented, followed by a broader discussion of each theme. Typical quotes are offered to illustrate themes, with further data available for each theme in Tables three to seven. Five key themes were identified, i.e., (1) physical and emotional impact of learning in practice, (2) lack of confidence in the process of assessment, due to inconsistency, (3) being prepared for practice, (4) impact of the practice supervisor/assessor, and (5) what might help. These are discussed below:

### 3.1. Physical and Emotional Impact on Practice Learning

Students felt anxious about meeting their proficiencies and transitioning into qualified roles, mainly due to a lack of support during placements. They recognized the need to be proactive in completing their requirements and getting sign-off from assessors. Students further reported that limited staffing often restricted opportunities to develop their skills.

*“…worry and confusion about us not having things signed off” Site 2.*

*“sink or swim … walk into as a qualified nurse that will either be the scaffolding around us, to support us up, or the bulldozer that destroys us” Site 3*

*“I feel like I have gone into year 3 drowning and I don't know anything, I do, but I just felt overwhelmed by it all and still now I do” Site 2.*

*“So, due to staffing, we couldn't really practice the proficiencies when we're supposed to be practicing” Site 2.*

The students at each site clearly described a physical and emotional impact on practice learning, the extent of which was not anticipated. There was evidence of emotive language throughout the three sites, and the sense of stress and frustration was clearly articulated. Students spoke of being worried, scared, lonely, and exhausted from having to “push” all the time. In this context, *“push”* denotes the continual physical, emotional, and mental effort students had to exert simply to cope and endure their practice learning. In essence, students survived, rather than thrived.

*“*…*I think you've got a really push for you know getting some of your proficiencies signed off… and I found that was quite challenging”* Site 2.

*“…they do put it a lot on students and say that this this pad is our responsibility to get signed and we must push for it to get signed and back”* Site 2.

### 3.2. Lack of Confidence in the Process of Assessment, due to Inconsistency

Students in this study reported inconsistencies in assessment practices across all sites. There was a lack of clarity and, at times, contradictions among assessors regarding how assessments should be conducted. For example, one assessor might consider a conversation sufficient to achieve a proficiency, while others required reflective discussions, single demonstrations, or multiple observed assessments. These variations caused confusion and frustration.

*“*…*There was lack of clarity, I've struggled to get signed off on my proficiencies*…*on placement like it's kind of like, because some people say you can get it signed off through having a conversation and then other people say you can't*…*”* Site 2.

*“Depending on your mentor. some of them are willing to, like, talk, they will let you talk through how you would do it and then pass you off on it”* Site 2.

*“Like some staff won't be happy to sign it off unless like you can do that independently or even teach it, whereas someone else will be happy with you just talking through it”* Site 1.

*“They were just happy to sign it off, they didn't speak to anyone or verify it or say have you met this proficiency yet*… *You were left to it, and then you just went and told them what you did, what you had done”* Site 3.

Students also noted differences in the level of interest shown in their learning by supervisors and assessors—some felt well-supported, while others were dissatisfied with the support received.

*“*…*just so knowledgeable, but he was also so encouraging at the same time”* Site 2.

*“She was, quite like*…*had high expectations, which I really appreciate now because it's helped me to be better”* Site 1.

*“Encouragement really helps build up confidence and appreciation as well. For some mentors will just sort of like disregard you and the effort that you put in. But when the ones that do appreciate and just like sort of notice your effort, it does you good and it does drive you to do better”* Site 3.

### 3.3. Being Prepared for Practice

The students described how they felt a lack of preparation for the practice environment, and how to learn in practice settings. Student described being unsure how to act or engage within the environment and that previous experience as care workers did not necessarily prepare individuals for the learning environment itself.

*“I'm worried that I don't have the knowledge that we should. Of the actual…not knowledge as in theoretical knowledge, being there as long as I have*… *Again, I'm a mature student myself. I have quite a large extensive caring background, I just feel underprepared. I feel like the clinical skills were quite rushed”* Site 1.

*“I wasn't prepared for placement as in what would be expected of me at all. How was I supposed to act, engage?”* Site 3.

*“*…*you walk into the placement areas and you are completely blind and it does take a while to settle in to*…*”* Site 2.

Students also spoke of some of the physical symptoms of feeling underprepared, such as feeling sick and lonely.

*“*…*we barely had any lessons so we didn't even have tutors. There was no one. It was very very lonely, and it was scary”* Site 3.

### 3.4. Impact of the Practice Supervisor/Assessor

The students were able to identify positive and negative aspects associated with the practice supervisors and assessors to their learning. The students were able to articulate the behaviors that facilitated learning and achievement of proficiencies (and those that were unhelpful). The ability of the practice supervisor and/or assessor to support the building of confidence among the students seemed to be an important factor to support practice learning. However, it seems that confidence could be fleeting and temporary, depending upon the situation and the people involved. Comments made by the students included the following:

*“Key people can make the difference, I had one assessor, and they thought I would know more than what I knew and that really knocked my confidence… There are many examples of this”* Site 1.

*“She's always said, I'm at the end of the phone with some words of encouragement to keep me going”* Site 2.

*“I think it depends who you get as a practice assessor, practice supervisor as well. And I found that you would go to some placements and they just didn't have the time for you…”* Site 2.

Some students reported that practice assessors and supervisors with laissez-faire attitudes negatively impacted their learning, often appearing disinterested, disengaged, or unaware of assessment criteria.

*“She just she had no interest whatsoever in sitting down, and that was something that obviously I've gone through the past three years, and I've got, like, so far, so I just took it upon myself*…*abandoned in a way”* Site 2.

*“I think some of our assessors were just very lenient”* Site 3.

*“…lack of preparation for PAs (Practice assessors): she didn't know how to use pad and she didn't wanna sign my pad…there was a lack of understanding in assessments…”* Site 2

### 3.5. What Might Help?

Students further identified what would be helpful to ensure that better support for practice learning and assessment took place. They described how being so focused on getting proficiencies signed off meant that often they forget to be present in the moment and simply enjoy their practice learning opportunity.

*“Sometimes it (proficiencies) can like take your focus away from like just being present on the ward…Actually just enjoying the placement”* Site 2.

Some students wanted more consideration of where each student was in their journey and more tailored support.

*“If they can judge like, where, we are and how we're feeling, then they can push us or like help us take a step back from something”* Site 2.

Other students were able to articulate that they wanted to answer questions, or be quizzed to provide a demonstration of their knowledge; again, the questioning ability of the practice supervisor or assessor appeared to be important in this respect; for example: one student spoke of wanting.

*“…To feel they have demonstrated competence actually be quizzed, be challenged”* Site 3.

*“It does build your confidence up a lot because when you're getting the answers right when you're explaining it to yourself or your supervisor, you think: Actually, I got that right that I do understand that next time if it comes up it gives you the validation that you need I think”* Site 2.

One student reflected on the value of teaching peers as a demonstration of personal mastery, highlighting how collaborative learning fostered mutual confidence among students. *“It's nice when we get to teach… because if we teach…, we know what we're doing as well. And then we can talk to the whole process. And then if they should have any questions and we don't know the answer, we can, that can add on to our own knowledge as well”* Site 1.

The group-based approach (the Collaborative Learning in Practice [Clip] model) was particularly well received, as it created an engaging environment that encouraged deeper interaction and collective learning.

*“I did really enjoy the CLip model and because it did feel a lot more free than when you were with your assessor… I think as well it does build your confidence up a lot…”* Site 2.

## 4. Discussion

The findings of this study reflect the complex challenges faced by third-year nursing students in practice-based learning under the NMC Future Nurse Standards (2018). These challenges offer critical insights into improving nursing education. This study underscores both physical and emotional challenges encountered by third-year nursing students in practice-based learning settings. These challenges highlight systemic issues, such as inconsistent assessment criteria, limited access to well-prepared supervisors, and the demanding nature of meeting proficiency standards. This discussion expands on these themes with existing literature to contextualize the findings.

In regard to the physical and emotional impacts faced by third-year nursing students in practice-based learning, there were notable physical and mental effects. Constantly having to “push and chase” for proficiency sign-offs adversely impacted their mental health. The phrase *“push”* denoted a continual physical, emotional, and mental effort students had to exert simply to cope and endure their practice learning. It conveys pressure, urgency, and possibly strain, suggesting that the learning environment may be intense or overly demanding. These findings align with studies on burnout and emotional exhaustion in nursing students, which emphasize the high psychological demands of clinical education [10]. Chronic stress from this “push and chase” dynamic as noted by Jacobsen et al. may lead to long-term mental health issues and contribute to attrition rates, though this study did not explicitly explore attrition. Students conveyed feelings of frustration, exhaustion, fear, and loneliness. The consistency of these emotions across three sites suggests such impacts are commonplace among nursing students. Wardrop et al. [11] suggest that structured peer mentoring could alleviate feelings of isolation, provide emotional support, and enhance learning experiences. Reflective practice sessions guided by experienced mentors could help students process these challenges and foster resilience [12].

The experience of learning in practice was often described as scary and lonely. The students had learned that to survive, they had to become hyper independent and drive the process of getting their documents signed off. However, the emphasis is on the signing off, rather than on the quality or depth of the learning. The focus of learning during final placement should be around building confidence and self-efficacy and facilitating the graduating nurse to take greater responsibility over time according to Rosli et al. [13]; however, it should be remembered that this relies on skilled support and facilitation and is only possible when those who are supporting the transitioning student are adequately prepared for their role [12]. It is beyond the scope of this study to explore the experiences of these students as they transition to becoming registrants, but early indications suggest they are already exhausted even before they become registrants. There was a lack of confidence in the processes carried out in the students' practice sites due to inconsistencies in the assessment processes. Students reported experiencing confusing and frustrating assessment processes characterized by notable inconsistencies across clinical sites. Assessment practices varied from reflective conversations with supervisors to repeated skill demonstrations before proficiency sign-offs. Such variability mirrors findings by Hanson et al. [2], who highlight that ambiguous assessment standards increase student anxiety and undermine confidence in their competencies. The inconsistent assessment approaches left students uncertain about the validity and depth of their learning. Some supervisors were perceived as lenient, approving proficiencies with minimal evidence, while others imposed more rigorous demands. Such discrepancies risk reducing assessments to procedural tasks rather than authentic evaluations of competence [4]. Many participants expressed self-doubt, questioning whether the leniency of their assessments accurately reflected their abilities, a concern that aligns with the imposter syndrome frequently observed in nursing education [9, 10].

Additionally, students noted that some supervisors and assessors lacked familiarity with program requirements and documentation, which hindered meaningful teaching and assessment. Conversely, instances where supervisors rigorously tested students' knowledge and provided constructive feedback were highly valued, as these interactions enhanced students' confidence and preparedness. Reflective dialog with experienced mentors who have time to teach is crucial for increasing confidence [14]. However, this study's findings suggest reflective discussions were often fleeting and superficial. Robust research into the exact nature of these practice-based reflective discussions could further inform improvements.

Preparation for professional practice is vital, with notable implications for students' confidence, competence, and overall success. This study revealed key challenges faced by students during their transition from theoretical learning to practical application. Historically, newly qualified registrants have reported feeling unprepared for their roles as teachers in practice environments [15–17]. Findings from this study suggest that despite changes to preregistration education standards in the United Kingdom, readiness for supervisory roles remains questionable. While students have historically been precluded from undertaking supervisory roles according to Hanson et al. [2], participants in this study offered few examples of teaching or supporting others, despite being “signed off” in these proficiencies. More research is needed to understand factors contributing to excellence in supervisory roles in practice settings.

Several students felt inadequately prepared for clinical placements, particularly in practical skills, often feeling rushed and concerned about their competencies. These concerns align with findings from Irvine and Martin [18] that gaps between theoretical instruction and clinical application often leave students struggling to meet workplace demands. Similarly, Halton et al. [19] emphasize the importance of embedding practical simulations in healthcare curricula to bridge these gaps effectively.

Transitioning from prior roles to healthcare education posed additional difficulties. Some participants reported unclear expectations regarding their transition from students to employed nurses, reflecting findings by Ricotta et al. [20] that role transitions are frequently hindered by unclear expectations and insufficient preparatory training. Despite these challenges, students recognized the necessity of taking initiative to overcome barriers. Jacqueline [21] highlights the importance of fostering self-directed learning in healthcare education but caution against overburdening students with responsibility. Structured support systems are critical to balancing self-directed learning with adequate guidance. These findings underscore the need for healthcare education programs to integrate comprehensive preparatory strategies, including preplacement resources, structured mentorship, and experiential learning opportunities.

Students' experiences were strongly influenced by their practice supervisors' capabilities. Inconsistent supervisor engagement led to varied learning outcomes. While some supervisors provided thorough guidance and constructive feedback, others were described as disengaged or uninformed about assessment criteria. This variation mirrors the observations of Logina and Traynor [12], who argue that supervisors' understanding of their roles greatly impacts students' learning experiences and professional preparedness. Overstretched supervisors often lack time and resources for meaningful mentorship, leaving students feeling isolated and underprepared [13]. Further, this lack of structured support discourages students from fully engaging in clinical learning opportunities, which is critical for their development as future supervisors and educators [22].

Positive relationships between students and clinical supervisors foster a sense of belonging, enhancing placement quality and learning outcomes. This study reconfirms the importance of empathetic and knowledgeable practice supervisors in facilitating meaningful learning experiences. However, many supervisors described in this study appeared overstretched or disinterested in teaching, echoing findings from Wardrop et al. [11]. Interestingly, while practice supervisors and assessors were frequently mentioned, other forms of support, such as practice education facilitators (PEFs), were notably absent. The PEF network may not have been visible or accessible to these students, or they may not perceive it as valuable, reflecting findings from Mathisen et al. [4].

### 4.1. What Might Help?

The transition to practice in healthcare education is a critical phase where students bridge theoretical learning with real-world applications. The importance of creating safe learning environments has long been recognized according to Kilminster et al. [23]. Feedback from students revealed a strong desire for better support mechanisms to enhance their learning experience. Students frequently expressed the desire to move beyond the administrative focus on signing off competencies, which often detracted from their ability to engage fully in the practice environment. Levett-Jones and Belongingness [22] emphasize the significance of creating learning environments that foster both competence and enjoyment, ensuring students can immerse themselves in placements to reflect on and internalize their experiences.

#### 4.1.1. Individualized and Tailored Support

A recurring theme in the feedback was the need for tailored support. Individualized mentorship improves student confidence and competence, especially when supervisors are approachable and adaptable to varying learning stages [24].

#### 4.1.2. Collaborative Learning Models

Students valued opportunities to work collaboratively and to receive feedback from diverse sources using models, such as the CLiP, which have been proven to promote critical thinking, teamwork, and peer learning [25]. Additionally, students appreciated opportunities to teach others as Archer [26] noted that teaching peers or junior colleagues enhances knowledge retention and confidence.

#### 4.1.3. Holistic Feedback Mechanisms

The importance of receiving feedback from multiple sources was another critical recommendation whereby the students found it helpful in building their skills and confidence. Multisource feedback is widely recognized for its ability to provide a more comprehensive evaluation of student performance and foster a sense of validation [26].

### 4.2. Strengths and Limitations

Strengths: This multisite qualitative study provides timely insights into the first cohort trained under the NMC Future Nurse Standards (2018). The use of hermeneutic phenomenology enabled rich exploration of student experiences. Rigorous methods, including data saturation and member checking, strengthened the study's credibility. Findings offer actionable recommendations for improving assessment consistency, supervisor training, and student support.

### 4.3. Limitations

The qualitative design and small sample size limited generalizability. The study lacked perspectives from supervisors or assessors, and long-term impacts on transition to practice were not explored.

## 5. Conclusions

This study provides detailed insights into practice-based learning experiences for the September 2020 cohort across three HEIs in England. Students identified factors enabling and hindering the achievement of NMC Future Nurse Standards (2018). A relentless focus on securing sign-offs overshadowed other aspects of learning, requiring significant energy and assertiveness. Assessment processes lacked clarity and consistency, leading to frustration, anxiety, and a lack of confidence. Learning in practice was often fraught with fear and loneliness, indicating systemic issues. Practice supervisors and assessors were identified as crucial to success, yet many remained overstretched, leaving practice learning neither fully understood nor adequately prioritized. Based on the findings of this study, the following systemic improvements are recommended to enhance practice-based learning experiences and address the identified challenges:

• Standardize assessment processes by developing clear, consistent guidelines for proficiency assessment to eliminate variability and ambiguity across clinical placements. This can include standardized checklists, uniform training for assessors, and detailed documentation templates. In addition, establish mechanisms for regular calibration of assessors to ensure alignment with NMC standards.
• Improve student preparation for practice by introducing immersive preplacement workshops and simulations to help students transition smoothly from theoretical learning to clinical practice. Additionally, developing tailored resources, such as digital orientation modules or detailed preplacement briefings, to familiarize students with specific clinical settings.
• Encourage collaborative learning models by expanding the use of models, such as CLiP, enabling students to work in groups, share experiences, and learn collectively. Integration of teaching opportunities for students will help to enhance their confidence and reinforce their learning through peer education.

The study recommends that future research focuses on longitudinal tracking of student nurse cohorts to examine the long-term impacts of the NMC 2018 standards. This approach would help ensure that practice-based learning remains both effective and meaningful, ultimately supporting the development of competent and confident nursing professionals.

## Figures and Tables

**Table 1:** Sample and recruitment.

Education institution	Total nursing students' population	Student sample
Site one	358 students	6 child field students and 1 adult field student
Site two	475 students	6 students (dual program, i.e., adult/mental health 1 child field student)
Site three	516 students	3 adult field students
Total no. of students	1349	17 students

**Table 2:** Topic guide for interviews.

Questions	Possible prompts
There are many standards of practice that you have to meet during your practice placements. What has been your experience of completing the Nursing and Midwifery Council Future Nurse Education Standards (2018) for you? *Lead Q: “we are interested in your experience of practice learning, and how you managed to meet the requirements of the PAD during the pandemic”* *Did it feel like you were “catching up”?* *As you moved between year 2 and year 3 did your feelings about practice learning change?*	How did things change, in your experience, because of the pandemic? How did you find meeting the requirements of your PAD? Which were the trickier things to do: • Proficiencies? • Episodes of care? • Reflections • Medication • Community care/close-to-home care? • Addressing skills more aligned to the other fields of practice Who or what was helpful to you? What did they do that made the difference? In an ideal world, what would a facilitative experience look like? [where you could meet all the standards]

What aspects of practice-based learning do you prioritize? And why? *Lead Q: “Are there any aspects of practice learning that you tend to prioritise?”* *Can you tell us more about this?*	What did you see as the most important things to attain? Why? Which skills and proficiencies did you feel were fundamental to your future role as a nurse? Where there any that you wondered why you needed to do them?

How have you been prepared for your own role as a practice supervisor, once you are qualified? *Lead Q: “can you tell us about opportunities you've had to supervise others, and how ready do you feel to take this on, on qualifying”*	What were your observations of your practice supervisors? Can you imagine yourself doing this role soon? What do you think that you will do well? What is the most important skills of a practice supervisor? Do you feel that you will have this skill by the end of the course? In an ideal way, what would the best practice supervision look like? Some possible prompts: • Prescriber ready? • Supervision models? [clinical supervision/CLiP, etc.] • On a scale of 1–10—how ready do you feel to take on the role of PS?

Anything else?	

Summarize, clarify, and thank	

## Data Availability

All the data collected from this study are available upon request from the authors and have been anonymized for participant protection.
